# Sulbactam–Durlobactam for Carbapenem-Resistant *Acinetobacter baumannii–calcoaceticus* Complex

**DOI:** 10.3390/pathogens15040449

**Published:** 2026-04-21

**Authors:** Francesco Nappi

**Affiliations:** Department of Cardiac Surgery, Centre Cardiologique du Nord, 93200 Saint-Denis, France; francesconappi2@gmail.com

**Keywords:** sulbactam–durlobactam, carbapenem-resistant *Acinetobacter baumannii*, *Acinetobacter baumannii* infection, multidrug-resistant *Acinetobacter baumannii–calcoaceticus* complex, ventilator-associated bacterial pneumonia, hospital-acquired bacterial pneumonia

## Abstract

Carbapenem-resistant *Acinetobacter baumannii* infections pose a significant challenge due to their severity and the poor prognoses they often result in, particularly in cases where there are risk factors present. The United States (US) Centers for Disease Control and Prevention (CDC) identified carbapenem-resistant *Acinetobacter baumannii* (CRAB) infections as a threat to human health. The World Health Organization (WHO) has classified it as a top priority for research. In 2023, the US FDA approved sulbactam–durlobactam for treating certain *A. baumannii* infections. As of 2024, this combination is designated as the preferred treatment strategy by the Infectious Diseases Society of America (IDSA) for infections due to carbapenem-resistant *A. baumannii*. In this therapeutic review, the preclinical and clinical data relevant to this regulatory decision were analyzed. This in-depth analysis will provide a comprehensive overview of the complex subject matter. It should be observed that carbapenem-based combination therapy is indicated for carbapenem-resistant *A. baumannii*.

## 1. Introduction

The genus Acinetobacter consists of Gram-negative coccobacilli that generally cause infections in healthcare facilities. The *Acinetobacter baumannii–calcoaceticus* complex (ABC) is a predominant isolate within this genus. Community-acquired infections are uncommon, but there is a risk for patients with comorbidities, elderly patients, diabetes mellitus, chronic lung or renal disease, malignancy, or impaired immunity. The most common sites of infection encompass the bloodstream, skin/soft tissue/surgical wounds, ventilator-associated pneumonia, orthopedic or neurosurgical procedures, and the urinary tract. *Acinetobacter* species are inherently resilient to numerous antimicrobials and possess a noteworthy capacity to acquire novel resistance determinants via plasmids, transposons, integrons, and resistance islands [1].

Since the 1990s, there has been a dramatic escalation in the prevalence of antimicrobial resistance (AMR) among ABC. The global spread of multidrug-resistant (MDR)-ABC strains indicates the dissemination of a few clones between hospitals, geographic regions, and continents. Excessive antibiotic use amplifies this spread. It is important to note that many strains are inherently resistant to all available antimicrobials, with the exception of colistimethate sodium and certain tetracyclines (minocycline or tigecycline). This presents a substantial challenge because some infections are currently untreatable with the available antimicrobial agents. AMR poses a serious threat to the effective treatment and prevention of ABC infections. To address the challenge of environmental contamination with MDR-ABC, it is imperative to implement comprehensive infection control measures and to categorize infected patients into designated groups. It is imperative that we implement deliberate antibiotic strategies to curtail the propagation of MDR-ABC. Optimal therapy will most likely involve the combination of existing antibiotics with new classes of antibiotics [1].

*Acinetobacter baumannii* is classified as a high-risk nosocomial bacterium due to its ability to cause life-threatening infections in healthcare facility patients and its extensive array of resistance mechanisms to the most commonly prescribed antibiotic medications. *A. baumannii* has been associated with a variety of health concerns, including respiratory tract infections (e.g., ventilator-associated bacterial pneumonia (VABP) and hospital-acquired bacterial pneumonia (HABP)), chronic and injury-related wounds, and bloodstream infections in individuals with centrally placed intravenous catheters. It is unfortunate that this organism is often found to have an extreme drug resistance (XDR) phenotype. Therefore, it is imperative that effective therapy be initiated at the earliest possible opportunity, as this is a significant clinical challenge [2].

*Acinetobacter* spp. are known to exhibit virulence derived from their ability to evade rapid clearance by the innate immune system. This process leads to an accumulation of bacteria, which in turn activates a pathway involving lipopolysaccharide (LPS)-Toll-like receptor 4 (TLR4). This cascade of events results in the onset of sepsis. Capsular polysaccharide plays a critical role in the virulence process, enabling immune evasion. Conversely, LPS has been identified as a trigger for septic shock. However, the primary factor influencing clinical outcomes is antibiotic resistance. The prompt administration of effective therapy is crucial for improving survival outcomes, with a threefold reduction in 30-day mortality being achieved [3]. Furthermore, the presence of MDR and extensively drug-resistant (XDR) *A. baumannii* infections in pediatric patients poses a significant threat, given the high mortality rate associated with such infections. In such cases, there is a significant risk of death, primarily attributable to infections of the bloodstream and central nervous system. Acute kidney injury is a serious condition that can be fatal [4].

**Figure 1:**
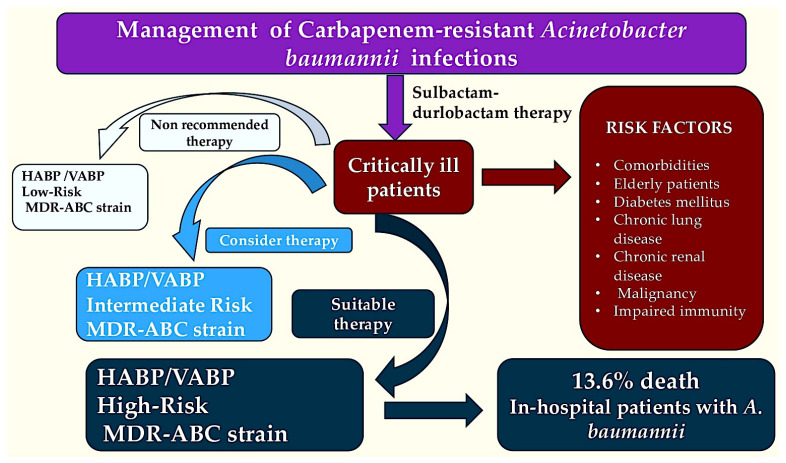
The figure illustrates the management of carbapenem-resistant *Acinetobacter baumannii* infections. The risk of MDR-ABC strains in critically ill patients is dependent on the specific risk factors they present. The decision regarding the implementation of sulbactam–durlobactam therapy is informed by the severity of the condition and the presence of risk factors. The light blue, dark blue, and blue colors of the boxes provide a clear indication of the degree of risk associated with developing an MDR-ABC strain in the event of HABP/VABP.

Estimates indicate that 13.6% of in-hospital patients with infections due to *A. baumannii* die; however, a significantly elevated mortality percentage of 33.6% has been recorded in ICU patients with bloodstream infections. A number of factors have been shown to be significant predictors of inpatient mortality. These include various existing conditions, the infectious source, the underlying illness, and inappropriate treatment. Analysis shows higher inpatient mortality rates for MDR *A. baumannii* versus non-MDR *A. baumannii* and carbapenem-resistant (CRAB) versus non-CRAB infections. Research shows longer average length of stay for MDR *A. baumannii* versus non-MDR *A. baumannii* and for CRAB versus non-CRAB [5]. Lodise et al. [6] found that, in a multicenter US study, *A. baumannii* was detected in a clinical culture in 1% of adult hospital admissions. Over a third of cases of *A. baumannii* infection in hospital were of the CRAB type, with the highest rates per 100 infection-related hospital encounters observed in the most centralized US regions. Clinicians should be aware of the potential for *A. baumannii* to be a pathogen in patients in regions where the incidence of the bacterium is increasing. It is not surprising that there are significantly worse clinical outcomes for patients with infections due to CRAB compared to those with susceptible infections. This is because there are delays in the administration of appropriate antibiotics and the treatment options available are limited [7]. Figure 1 illustrates the management of carbapenem-resistant *A. baumannii* infections in patients hospitalized in the intensive care unit.

The issue is being made worse by the fact that more and more *A. baumannii* bacteria are resistant to carbapenems. In North America, the rate rose from 11.2% to 40.3% between 1997 and 2016 [8,9], and has now passed 80% in some regions of Latin America, Eastern Europe and the Asia-Pacific region [10,11]. In light of these developments, the United States Centers for Disease Control and Prevention has identified CRAB as a pressing concern for public health [12,13], and the World Health Organization has classified CRAB as a priority category I pathogen, with the utmost urgency for investigation and the development of new therapeutic interventions [14].

Sulbactam–durlobactam has been granted market authorization in the United States by the FDA in 2023 for the treatment of HABP and VABP caused by antibiotic-resistant strains of *A. baumannii*. This therapeutic review has been conducted to offer a comprehensive overview of the role of sulbactam–durlobactam in the therapeutic management of infections caused by CRAB. Furthermore, a range of challenging and emerging subjects have been reviewed.

### Search Strategy

The therapeutic review was designed and the database investigation was conducted in June 2025. A comprehensive investigation was conducted using the following search terms in MEDLINE, Embase, and the Cochrane Library: sulbactam–durlobactam; carbapenem-resistant *Acinetobacter baumannii*; *Acinetobacter baumannii* infection; multidrug-resistant *Acinetobacter baumannii–calcoaceticus* complex; ventilator-associated bacterial pneumonia; hospital-acquired bacterial pneumonia. The aforementioned terms were combined with additional search terms, including “World Health Organization”, “Central Disease Control”, and “Federal Drug Administration”. The selection of publications concentrated on material published from 2004 to 2025, with an emphasis on the recent literature. In addition, the reference lists of articles identified by this search strategy were thoroughly searched, and relevant articles were selected. The search was focused on identifying data from the following sources: randomized controlled trials (RCTs), meta-analyses and observational cohort studies. The selection of review articles was made to furnish readers with more detailed background references and further reading (Table 1).

## 2. Treating Carbapenem-Resistant *Acinetobacter baumannii*: Microbiological Rationale and Clinical Trial Evidence

The management of CRAB infections poses significant challenges due to a number of factors.

Firstly, it is most frequently detected in patients’ respiratory samples or wounds. However, it is not always clear if a CRAB strain isolated from a respiratory or wound culture in medically complex patients represents a colonizing organism, or if it is a true pathogen. This can lead to uncertainty about the need for antibiotic therapy. Therefore, it is challenging to determine whether suboptimal administration of antibiotic treatment or inherent host factors are the cause of inadequate clinical results with CRAB infections.

Secondly, in cases where *A. baumannii* has developed carbapenem resistance, it typically possesses resistance to most other anticipated antibiotic treatments for wild-type *A. baumannii,* significantly reducing available treatment alternatives. The production of OXA carbapenemases (e.g., OXA-23, OXA-24/40) has been identified as a mechanism that contributes to resistance to β-lactams, encompassing carbapenems and sulbactam [15,16]. CRAB isolates frequently yield the production of further serine β-lactamases (e.g., Aci-netobacter baumannii-derived cephalosporinases [ADCs]), thus hindering the effectiveness of common β-lactam medications. Sulbactam resistance is primarily caused by the activity of β-lactamases, but also by mutations that affect PBPs (PBP1a/1b, and PBP3) [17,18,19]. The scientific community generally agrees that aminoglycoside altering enzymes, also known as 16S rRNA methyltransferases, often prevent the use of aminoglycosides as a treatment for CRAB [20,21,22]. Research has demonstrated that mutations in the regions of chromosomes that determine resistance to quinolones generally result in resistance to fluoroquinolones [21].

Despite extensive research in clinical trials investigating optimal treatment regimens for CRAB infections, current data is insufficient to definitively support specific agents with CRAB activity or the additive benefit of commonly used combination regimens for CRAB infections. This guideline document provides specific direction on the management of moderate-to-severe CRAB infections.

As outlined in Table 2, the suggested doses of beta-lactam antibiotics for the treatment of antimicrobial-resistant infections involving both *A. baumannii* and non-*A. baumannii* (see Section 3) in adult patients are detailed, under the assumption of normal renal and hepatic functioning.

### 2.1. General Approach for the Treatment of Infections Caused by CRAB: A Focus on IDSA Recommendation

Sulbactam is classified as a penicillanic acid sulfone beta-lactamase inhibitor (BLI), a category of pharmaceuticals. The laboratory tests on the effects of sulbactam combinations with other antibiotic groups against clinical *Acinetobacter baumannii* isolates have shown very encouraging results.

For the treatment of CRAB infections, the use of an antibiotic therapy comprising a sulbactam-containing agent is strongly advised. The most effective treatment option is sulbactam–durlobactam in combination with a carbapenem (e.g., imipenem–cilastatin or meropenem). If sulbactam–durlobactam is unavailable, a suitable alternative is a high-dose ampicillin–sulbactam regimen, administering a total daily dose of 9 g of the sulbactam component in conjunction with at least one other agent, such as polymyxin B, minocycline, tigecycline, or cefiderocol.

The initial research project aimed to determine the in vitro interactions between sulbactam and various other antibiotics, including ceftazidime, ceftriaxone, cefepime, ciprofloxacin, gentamicin, meropenem, tigecycline and colistin. The results indicated that sulbactam is a promising candidate for combination treatment regimens for MDR *A. baumannii* infections [23]. These interactions were interpreted according to the fractional inhibitory concentration (FIC) index.

Ten clinical isolates of *A. baumannii* were examined to determine the synergistic impacts of sulbactam when used with various antimicrobial combinations. There has been a significant decrease in minimal inhibitory concentration values. The sulbactam–ceftazidime–gentamicin combination was found to exhibit synergy (FIC index ≤ 0.5) and partial synergy (FIC index > 0.5 to <1). Furthermore, the minimal inhibitory concentration (MIC) values for both ceftazidime and gentamicin for five distinct strains decreased to levels below the established breakpoint, indicating a positive interaction between the two medications. Similarly, the MIC value of ciprofloxacin for six ciprofloxacin-resistant isolates was below the breakpoint for combination susceptible results. It is important to note that all strains were susceptible to colistin and tigecycline, with MIC values reduced in conjunction with sulbactam. While sulbactam and ceftriaxone combinations demonstrated synergistic and partial synergistic effects, all isolates exhibited resistance to ceftriaxone. The cefepime–sulbactam pairing demonstrated clear synergy in five isolates, partial synergy in one, and no effect in four. Meropenem and sulbactam demonstrated a partial synergistic effect (FIC index: >0.5 to <1) in three isolates, an additive effect (FIC index: 1) in one isolate, and an indifferent effect (FIC index: >1–2) in six isolates. The study definitively ruled out antagonism in any combination for clinical *A. baumanni* isolates [23,24,25,26,27].

The standard protocol for managing CRAB infections involves the administration of dual therapy, utilizing a combined regimen of at least two agents, until an appropriate clinical response is observed. This approach is informed by the limited data available on the effectiveness of individual antibiotic agents in treating such infections. In addition, it is generally recommended that at least one agent in the mixture is sulbactam-based. It is recommended that sulbactam-based treatment options include the administration of sulbactam–durlobactam in conjunction with either imipenem–cilastatin or meropenem.

In instances where sulbactam–durlobactam is unavailable, one alternative option is to administer a high-dose ampicillin–sulbactam combination, with a total daily dose of 9 g of the sulbactam component, as part of a comprehensive therapy regimen. In laboratory tests, animal studies, and clinical trial results, sulbactam has demonstrated its distinctive effectiveness in combating *A. baumannii* isolates [28,29,30,31]. Administration of high-dose sulbactam is recommended for combination therapy, despite the fact that only one of seven clinical trials found improvement in clinical results with combined antibacterial therapy for CRAB infections [28,32,33,34,35,36,37,38]. It is noteworthy that the clinical trial that demonstrated the efficacy of combination treatment was the only one that included high-dose ampicillin–sulbactam in the group receiving combination therapy [28]. In addition to high-dose ampicillin–sulbactam, there are other antibiotics that can be used in combination with this antibiotic, including polymyxin B, minocycline, tigecycline, and cefiderocol. It is not advisable to use fosfomycin and rifampin together as part of combination therapy [35,37,38].

Two major clinical trials have not indicated a proven advantage to patients of high-dose, extended-infusion carbapenem treatment administered in conjunction with colistin for the management of CRAB infections [33,34]. As a result, meropenem or imipenem–cilastatin are not recommended as standard treatment options for CRAB infections. However, they can be prescribed in conjunction with sulbactam–durlobactam. Nebulized antibiotics are not recommended as an additional treatment for CRAB pneumonia. The absence of evidence supporting their efficacy in clinical trials [39,40,41] and concerns regarding their distribution in the lungs have raised safety concerns, including the potential for respiratory complications such as bronchoconstriction [42,43,44,45].

As illustrated in Table 3, the 2024 CLSI guidelines delineate the antibiotic susceptibility cut-off values for *A. baumannii* and *non* *-A. baumannii* Gram-negative organisms, in addition to antibiotic combinations, as stipulated within the IDSA AMR guideline compendium.

**Table 3:** 2024 CLSI guidelines on susceptible breakpoints for *A. baumannii* and non-*A. baumannii* Gram-negative organisms and antibiotic combinations, as outlined in the IDSA AMR guideline document *.

Antibiotic	*Pseudomonas aeruginosa* (μg/mL)	Enterobacterales (μg/mL)	Carbapenem-Resistant *Acinetobacter baumannii* (μg/mL)	*Stenotrophomonas maltophilia* (μg/mL)
**Ampicillin–sulbactam**	- - -	- - -	≤8/4	- - -
**Ceftazidime–avibactam**	≤8/4	≤8/4	- - -	- - -
**Ceftolozane–tazobactam**	≤4/4	≤2/4	- - -	- - -
**Cefepime**	≤8	≤2 ^#^	- - -	- - -
**Cefiderocol**	≤4	≤4	≤4	**- - -**
**Imipenem–relebactam**	≤2/4	≤1/4	- - -	- - -
**Imipenem**	≤2	≤1	- - -	- - -
**Meropenem–vaborbactam**	- - -	≤4/8	- - -	- - -
**Meropenem**	≤2	≤1	- - -	- - -
**Sulbactam–durlobactam**	- - -	- - -	≤4/4	- - -

Abbreviations; CLSI, Clinical and Laboratory Standards Institute; * for full information on the interpretation of antibiotic susceptibility testing, please consult [46,47] Wayne, PA. The CLSI M100 document is updated annually and the susceptibility criteria are subject to change in 2025. ^#^ Isolates with MICs ranging from 4 to 8 μg/mL demonstrate susceptibility, exhibiting a dose dependence.

### 2.2. The Role for Sulbactam–Durlobactam as a Therapeutic Option for the Treatment of CRAB-Induced Infections

Durlobactam is an effective inhibitor of β-lactamases, specifically Class A enzymes (e.g., TEM-1), Class C enzymes (e.g., AAC), and Class D enzymes (e.g., OXA-24/40, OXA-23). It demonstrates potent inhibitory activity against these types of enzymes. The inhibitor does not affect Class B MBLs, for example NDM, the prevalence of which is increasing in isolates from the US, and which are produced in low quantities by CRAB isolates in the US.

The prevalence of Class B MBL is however much higher in other regions of the world, with at least 5% of CRAB isolates in Latin America containing a blaNDM between 2017 and 2019 and current estimates being likely to be higher [46,47]. Research has shown that durlobactam can reduce the likelihood of sulbactam hydrolysis by interacting with and inhibiting Class A, C, and D β-lactamases. This allows sulbactam to successfully reach its PBP receptors [48] and be effective. The dosage of sulbactam–durlobactam prescribed is 1 g of sulbactam and 1 g of durlobactam (2 g in total) administered every 6 h over a 3 h period as a continuous infusion [49] (Table 1). The dosing plan successfully attains PK/PD target goals in over 90% of *A. baumannii* isolates with sulbactam–durlobactam MICs of ≤4/4 μg/mL, as defined by both the FDA and the Clinical and Laboratory Standards Institute (CLSI), as documented in reference [49].

A clinical trial was conducted to investigate the efficacy of sulbactam–durlobactam in patients suffering from pneumonia or bloodstream infections resulting from *A. baumannii* [33]. Patients were divided into two groups for the study’s purposes: the first group was given sulbactam–durlobactam, while the second group was given colistin. All patients also received imipenem–cilastatin, dosed at 1 g of imipenem every 6 h. The primary outcome of 28-day mortality was evaluated for 125 patients with CRAB infections. The mortality rate was 19% (12/63) in the sulbactam–durlobactam group and 32% (20/62) in the colistin group, which met the pre-specified non-inferiority criteria. Secondary outcomes demonstrated that sulbactam–durlobactam also exhibited superior performance, achieving clinical cures in 62% of cases compared to 40% with the older treatment, and microbiological responses of 68% against 42% for the older treatment, as well as a lower risk of nephrotoxicity of 13% against 38%. It is important to note that the comparative treatment arm in this trial (i.e., colistin plus imipenem–cilastatin) is not a recommended course of action for CRAB infections.

The research indicates that imipenem–cilastatin may offer a clinical benefit that is comparable to that of sulbactam–durlobactam. Studies shows that the co-administration of sulbactam–durlobactam and imipenem–cilastatin reduces the MIC of sulbactam–durlobactam by 1- to 2-fold [50,51,52]. It is plausible that the observed potential benefit is associated with the supplementary PBPs that are addressed by multiple β-lactams. For instance, sulbactam demonstrates a higher binding affinity for PBP1 and PBP3, while imipenem exhibits a stronger binding affinity for PBP2 [51,53]. Research has shown that both sulbactam and imipenem have an increased ability to effectively reach their PBP receptors when protected by durlobactam. In addition, there is a possibility that imipenem functions as a substrate for OXA-carbapenemase-mediated hydrolysis. This could result in increased sulbactam reaching its PBP receptors. A study using a specially designed apparatus to model infected tissue found that administering a carbapenem in combination with sulbactam–durlobactam resulted in a significant reduction in bacterial growth [54]. Unfortunately, there is currently no clinical data available on the benefits of sulbactam–durlobactam for the treatment of CRAB infections in the absence of imipenem–cilastatin. According to the available in vitro data, it is recommended that imipenem–cilastatin be prescribed as an additional treatment to sulbactam–durlobactam. Meropenem is a suitable alternative to imipenem–cilastatin, given that they have similar PBP targets [51,52]. In cases where patients require long-term therapy (e.g., CRAB osteomyelitis), it may be appropriate to consider discontinuing carbapenem medication once clinical improvement has been achieved.

As the use of this agent in clinical practice increases, our understanding of the mechanisms of resistance to sulbactam–durlobactam will evolve. The current body of research indicates that elevated resistance to sulbactam–durlobactam is predominantly attributable to MBL enzymes or PBP3 mutants [50,55]. In the context of resistance to sulbactam–durlobactam (i.e., MICs ≥16/4 μg/mL), the panel advises consideration of non-sulbactam combinations that are optimally dosed (e.g., cefiderocol, minocycline, tigecycline, and polymyxin B). This is because sulbactam-based therapy is unlikely to offer significant therapeutic value (Table 2).

### 2.3. Ampicillin–Sulbactam: A Treatment for Infections Caused by CRAB

High-dose ampicillin–sulbactam has been proposed as a substitute for CRAB in the context of combined therapeutic regimens. This alternative is recommended only in cases where the availability of sulbactam–durlobactam is restricted.

Sulbactam is a highly effective drug that works by inhibiting the action of certain bacteria-killing enzymes. It has been shown to be particularly effective against *A. baumannii* strains when administered in high doses. Sulbactam has been shown to have distinct activity against *A. baumannii* strains in pharmacokinetics/pharmacodynamics (PK/PD) studies [24,25,56,57,58,59,60,61], animal models [27,62], and clinical outcomes data [28,29,30,31]. The expert committee advises the use of high-dose ampicillin–sulbactam (a total daily dose of 9 g of the sulbactam constituent) as a key element of combination therapy for CRAB infections. Table 1 presents an evaluation of existing PK/PD statistics and reveals that a daily sulbactam dosage of 9 g is expected to attain sufficient fT > MIC (regardless of whether the threshold is 40% or 60% fT > MIC) for *A. baumannii* strains with sulbactam MICs ranging up to 16–32 μg/mL (i.e., sulbactam-resistant strains) [63]. Ampicillin–sulbactam follows a 2:1 formula, with 3 g of the antibiotic consisting of 2 g of ampicillin and 1 g of sulbactam. A total day-to-day prescription of 27 g of ampicillin–sulbactam (which is equivalent to 9 g of sulbactam) should be given in the form of either repeated or ongoing intravenous infusions (for example, 9 g of sulbactam administered intravenously every 8 h over a 4 h period) [24,25,28,29,57,64].

Durlobactam is a highly effective inhibitor of Class A, C and D enzymes, which are often present in CRAB [55,65]. This allows for reduced dosages of sulbactam, which in turn can effectively achieve its PBP goals while being shielded by durlobactam. It should be noted that ampicillin–sulbactam does not offer the additional protective benefits of a durlobactam-like beta-lactamase inhibition mechanism.

Research indicates that less than 50% of CRAB strains are sensitive to ampicillin–sulbactam [66,67]. At this time, there is insufficient evidence to establish whether ampicillin–sulbactam at standard or high dosages has comparable effectiveness when treating CRAB infections resulting from pathogens that are vulnerable to ampicillin–sulbactam. The expert opinion is that high-dose ampicillin–sulbactam is the preferred option, due to its demonstrated ability to saturate sulbactam’s PBP target sites. This is particularly relevant given the likelihood that substantial quantities of the drug will be hydrolyzed by β-lactamases before achieving these target sites. Additionally, there are concerns about the reliability of conventional methods for ampicillin–sulbactam AST testing for CRAB, which may not accurately reflect true susceptibility, as evidenced by discrepancies in results obtained using AST methods other than reference broth microdilution [68,69]. Two meta-analyses have been conducted on both observational and clinical trial results for a variety of antibiotic combinations used to tackle CRAB infections [30,31]. A 2021 meta-analysis encompassing 18 relevant publications and 1835 patients revealed that ampicillin–sulbactam (a total dosage of at least 6 g of the sulbactam component) in conjunction with an additional agent emerged as the most impactful combination therapy, achieving the optimal outcome in terms of mortality reduction among patients acutely infected with CRAB [30]. A previous meta-analysis, published in 2017, encompassed a total of 23 observational research investigations and clinical trials, involving 2118 patients with CRAB infections [31]. The analysis concluded that sulbactam has the most significant effect on mortality in regimens involving sulbactam, polymyxin, or tetracycline (Table 2).

It should be noted that a minimum of five clinical trials evaluating mortality in patients with CRAB infections have included sulbactam in one of the treatment arms [70]. The mortality rates in the colistin-based treatment group were compared with those in the sulbactam-based group in these trials, and the results were as follows: 42% vs. 33% [71], 82% vs. 42% [72], 63% vs. 50% [28], 38% vs. 17% [73], 32% vs. 19% [32]. While statistical significance in terms of differences in survival was observed in only one of the trials, all trials indicate a decrease in survival in the sulbactam-based treatment group [72]. This supports the hypothesis that the addition of sulbactam to the standard therapeutic protocol may offer a benefit. After a thorough review of all available in vitro, animal, and clinical data, the panel has determined that high-dose ampicillin–sulbactam, in combination with an alternative agent, is a viable treatment option for CRAB infections, in instances where sulbactam–durlobactam is not readily accessible.

## 3. Discussion

### 3.1. IDSA’s Recommendation Is Unambiguous: High-Dose Sulbactam Is Highly Beneficial

Studies have shown that sulbactam displays selective, irreversible inhibitory effects on beta-lactamase enzymes from the Ambler Classes A and C. Regarding *A. baumannii*, sulbactam has been found to possess inherent antibacterial properties. Sulbactam is an effective antibacterial agent that targets the penicillin-binding protein 3 (PBP3) of the *A. baumannii* strain, with a lesser effect on PBP1a/1b. This medication has demonstrated a low propensity for developing resistance, with only a limited number of pbp3 mutants exhibiting high levels of resistance to sulbactam and reduced fitness [17]. In the absence of effective therapeutic interventions for severe CRAB infections, high-dose ampicillin–sulbactam has emerged as a critical component of targeted treatment regimens. Clinical guidelines strongly recommend its use [74,75,76] (Table 1).

However, it is important to note that carbapenem resistance is often linked to a decrease in the in vitro effectiveness of sulbactam [77,78]. Sulbactam’s clinical effectiveness is only determined when administered in conjunction with its inactivated counterpart, ampicillin. Therefore, it is imperative for medical professionals to recognize that the ampicillin–sulbactam minimum inhibitory concentration (MIC) (e.g., 2 when the MIC is 4/2 mg/L) corresponds to the sulbactam MIC. A recent study examined 10,749 *A. baumannii* strains for their sensitivity to ampicillin–sulbactam. The Clinical and Laboratory Standards Institute (CLSI) breakpoint of MIC ≤ 8/4 mg/L (i.e., 4 mg/L of sulbactam) was used to determine susceptibility, and 37.4% of the strains were found to be susceptible [77] (Table 2).

Nevertheless, in the group of 6541 tested isolates that were imipenem-non-susceptible, the cumulative rate of sulbactam sensitivity dropped significantly to 4.5%. The results of this study align with the low sulbactam susceptibility rates observed in CRAB isolates from patients with ICU-acquired pneumonia. The ATLAS dataset, which includes 2905 global meropenem-resistant (MIC > 8 mg/L) respiratory *A. baumannii* strains isolated from ICU patients between 2014 and 2021, indicates that only 5.7% of strains were found to be sensitive to sulbactam. The reported MIC50 and MIC90 values were 32 and ≥64 mg/L, respectively [78]. When considered as a whole, these observations indicate that the processes leading to diminished carbapenem susceptibility are also accountable for the diminished in vitro effectiveness of sulbactam. In the context of Class D carbapenem-hydrolyzing OXA enzymes, which are predominant in CRAB, particularly the OXA-23 subgroup, along with the corresponding Class A and C TEM-1 and ADC enzyme categories, there is evidence to suggest that these enzymes can effectively inactivate sulbactam. This has been implicated in the observed increases in MICs.

The issue under consideration is whether the standard or more aggressive administration of ampicillin–sulbactam can counteract this heightened MIC frequency in CRAB. The recent availability of PK/PD evidence has facilitated the answer to this question. The PK/PD profile of sulbactam in *A. baumannii* has been determined using both in vitro and in vivo (animal) models. The study’s objective was to determine the PK/PD index most closely associated with bacterial eradication and the concentrations required to attain antibacterial activity. A sulbactam-susceptible (sulbactam MIC 0.5 mg/L) strain was used to achieve this [79].

The findings from both the thigh and lung experiments indicate that fT > MIC is most significantly associated with the effectiveness of sulbactam. Further assessment was necessary to determine the optimal fT > MIC value for sulbactam, which is required to achieve bacterial stasis and 1-log10 kill. This assessment revealed that achieving target levels of 21.0% and 32.9% in the thigh test model and 20.4% and 24.5% in the lung test model was directly linked to the desired sulbactam activity concentrations. While this evaluation yielded valuable insights, its findings were limited by the use of a single, vulnerable model strain. More recently, the PK/PD of sulbactam in *A. baumannii* has been subject to rigorous evaluation in a murine lung model incorporating data from 21 clinical strains with sulbactam and meropenem minimum MICs spanning from 1 to 32 mg/L and 0.25 to >64 mg/L, accordingly [80].

Once again, the PK/PD index best linked to antibacterial effectiveness has been confirmed to be fT > MIC. However, further analysis has revealed a clear distinction in the magnitude of fT > MIC between 17 isolates deemed sensitive or intermediate to sulbactam or meropenem and four reported cases of resistance to both medications. The study revealed that 17 strains were found to be either resistant or intermediate to at least one medication. The median fT > MIC values of 11.6% and 21.2% were necessary to achieve stasis and 1-log10 kill with sulbactam, respectively. However, the four carbapenem-resistant strains with heightened sulbactam minimum inhibitory concentrations (>8 mg/L) required significantly elevated sulbactam fT > MIC levels of 50.6% and 60.4% to attain a comparable outcome. A Monte Carlo simulation (MCS) was conducted using these parameters with sulbactam PK models derived from 16 critically ill subjects with *A. baumannii* VABP [56].

In this particular MCS, a more conservative strategy was employed, setting a reduced threshold of 21.2% fT > MIC for isolates demonstrating susceptibility or intermediate resistance to sulbactam. Conversely, a more stringent objective of 60.4% was designated for sulbactam-resistant isolates [80]. The analysis indicates that both the conventional dosage (1 g administered every 6 h over 0.5 h) and the increased dosage (3 g administered every 8 h over 4 h) of sulbactam can attain a high probability of target achievement (PTA) of approximately 90% or higher, up to an MIC of 8 mg/L (sulbactam-intermediate). However, both regimens were unable to attain a PTA above 60% in sulbactam-resistant strains, largely due to the significant rise in the fT > MIC target when transitioning to this group. The Infectious Diseases Society of America (IDSA) has recommended high-dose sulbactam-based therapy as part of treatment plans for CRAB, regardless of sulbactam MIC. However, it is important to note that this was only the recommended course of action for CRAB treatment prior to the introduction of sulbactam–durlobactam. The most recent PK/PD results, when viewed in conjunction with the current lack of clinical data supporting sulbactam use in cases of sulbactam resistance, underscore the pressing need for more effective alternatives.

### 3.2. Could Sulbactam–Durlobactam Be the Solution?

Durlobactam is a new antibiotic drug that has been developed to target Gram-positive and Gram-negative bacteria. It is part of a group of drugs called diaza-bicyclooctanone (DBO), BLI, and was created by modifying the structure of avibactam. This modification was intended to enhance its inhibitory effect on Class A and C enzymes, broaden its spectrum of inhibition to include a wider range of Class D enzymes, and improve its ability to penetrate Gram-negative cell membranes [81]. Durlobactam has been shown to demonstrate 10-fold decreases in IC50 (half maximal inhibitory concentration) when evaluated alongside avibactam. Notable reductions were observed for both KPC-2 and AmpC. In addition, a significant 100-fold decrease in IC50 has been observed when comparing durlobactam to OXA-24. The translation of strong Class D antibacterial efficacy into in vitro bacterial strain proliferation inhibition is corroborated by >4-fold minimal inhibitory concentration (MIC) reductions observed in isogenic strains of *A. baumannii* overexpressing OXA-23 and OXA-24 when durlobactam was administered independently to piperacillin, meropenem, and sulbactam at a fixed concentration of 4 mg/L [81].

Durlobactam has been demonstrated to selectively inactivate PBP2 in *A. baumannii*, leading to alterations in cell structure. However, it does not demonstrate standalone antibacterial efficacy against this strain, with an MIC50 of >64 mg/L [81,82]. Durlobactam has been shown to effectively reinstate the efficacy of sulbactam through the process of beta-lactamase suppression. In a study of 5032 global clinical isolates, it achieved a 32-fold reduction in the sulbactam MIC90, from 64 to 2 mg/L. It demonstrated a 98.3% degree of sensitivity at the 4/4 mg/L cut-off point [48]. For all antimicrobial-resistant strains of *A. baumannii*, including those that are resistant to carbapenems and colistin, sulbactam–durlobactam demonstrated over 96% susceptibility. Further research is necessary to comprehensively understand the resistance processes involved in sulbactam–durlobactam non-susceptibility. However, the analysis of sulbactam–durlobactam-non-susceptible strains has identified two key processes. Research has revealed that durlobactam exhibits substandard inhibitory activity against Class B metallo-β-lactamases (MBLs).

It has been observed that the expression of New Delhi MBL-1 (NDM-1) is associated with a high level of resistance to sulbactam–durlobactam, with observed MICs greater than 32/4 mg/L [55,65,83]. Though MBLs are present in only a small percentage of *A. baumannii* strains in the United States, the threat posed by NDM-producing CRAB is significantly higher in Latin America and the Middle East [47]. Mutations in PBP3, which represents the key target site for sulbactam, have been identified as a frequent pattern of decreased sulbactam efficacy. Research indicates that the most frequent PBP3 mutations affecting sulbactam–durlobactam efficacy are A515V, Q488K and Y258H (co-produced), and T526S substitutions. While these mutations result in elevated MIC values, often rendering the organism non-susceptible, they are generally associated with lower MICs compared to MBL producers [84,85]. As molecular testing in in vitro studies has not yet been conducted on many non-susceptible strains, the precise molecular processes that underpin sulbactam–durlobactam resistance remain to be fully elucidated. However, there is some evidence to suggest that mutations in the gene responsible for expressing the AdeJ efflux pump may also result in a decrease in effectiveness [85,86].

The ATTACK trial evaluated the clinical efficacy of sulbactam–durlobactam. The trial population included patients with pneumonia or bloodstream infections due to CRAB. Patients were divided into groups to administer either 1 g (for both constituents) of sul-bactam–durlobactam intravenously (IV) every 6 h over 3 h, or 2.5 mg/kg of co-listin IV every 12 h for 7–14 days. All study participants received 1 g of imipenem via the intravenous route at 6-h intervals in combination with their assigned study medication. This combination therapy covers for the treatment of other potential Gram-negative pathogens, in addition to polymyxin monotherapy, which has been shown to cause relative discomfort in patients. The effectiveness of the treatment was evaluated in the microbiologically modified intention-to-treat group, which included participants with eligible infection types who received a dose of the study drug and had confirmed carbapenem-resistant *A. baumannii* strains that remained vulnerable to both sulbactam–durlobactam and colistin. The primary endpoint of 28-day all-cause mortality was evaluated in 125 patients, and sulbactam–durlobactam was found to be non-inferior to colistin, with a mortality rate of 19.0% as opposed to 32.3% (treatment difference −13.2%; 95% CI −30.0% to 3.5%). Sulbactam–durlobactam has also been shown to lead to considerably higher rates of clinical cure (62% versus 40%; a 22% treatment difference, 95% CI 2.9% to 40.3%). Furthermore, there were lower rates of acute kidney injury (13% versus 38%; *p* < 0.01) in the sulbactam–durlobactam group compared with patients treated with colistin [32].

The relatively limited number of patients included in the ATTACK study, coupled with the absence of additional clinical trial data, may temper the degree of confidence in the efficacy of sulbactam–durlobactam. However, it is noteworthy that cefiderocol did not demonstrate comparable outcomes in the subgroup of patients with CRAB infections in the CREDIBLE-CR trial, who were predominantly treated with best-available therapy (BAT), which often includes colistin-based regimens. It is worth noting that the 28-day mortality rate was found to be 38% in the cefiderocol group, as opposed to 18% in the BAT group. While supporting evidence is limited, the data available does offer the most compelling proof to this point of a clinical advantage for sulbactam–durlobactam in treating CRAB infections, supporting its primary use in such cases. Accordingly, IDSA guidelines now recommend sulbactam–durlobactam as the first-line treatment for severe CRAB infections, with the proviso that it should be given with a carbapenem, as reflected in the design of the clinical trials [74].

### 3.3. CRAB Treatment with Sulbactam–Durlobactam in Combination with Carbapenem

Although it has been shown to be effective against CRAB in in vitro tests, sulbactam–durlobactam has a relatively limited effectiveness of antibacterial activity when it comes to other Gram-negative pathogens. It is likely that the direct PBP2 inhibition exhibited by durlobactam contributes to its inherent capacity to combat a range of bacterial strains, including *Klebsiella pneumoniae*, *Escherichia coli*, *Enterobacter cloacae*, *Stenotrophomonas maltophilia* and *Citrobacter* spp., thereby broadening the antibacterial efficacy of sulbactam [81]. Despite the encouraging results from in vitro studies, sulbactam–durlobactam has efficacy in combating *Pseudomonas aeruginosa*, a leading cause of hospital-acquired pneumonia. It should be noted that there is currently no compelling data demonstrating the efficacy of PBP2 inhibitors for treating severe nosocomial infections. Given the ineffectiveness of sulbactam–durlobactam in combating *P. aeruginosa*, it was not included as a standalone treatment in the ATTACK trial. This is particularly relevant in light of the fact that the occurrence of polymicrobial infections in recently conducted clinical trials of patient populations with hospital-acquired pneumonia has ranged from 20% to 40% [32,86,87,88,89].

While it is evident why a dual-therapy regimen with imipenem is included in clinical trials, clinicians must determine if the recommendation in the IDSA guidelines to administer a carbapenem in combination with sulbactam–durlobactam in clinical practice is appropriate. The absence of more robust data based on multicenter RCTs highlights the necessity for further in-depth studies. From a mechanistic perspective, there is a clear rationale for increased efficacy with the imipenem–sulbactam–durlobactam association. This is due to the complementary PBP binding of sulbactam (primarily PBP3 and, to a lesser extent, PBP1b) and imipenem (primarily PBP2 and, to a lesser extent, PBP1a). This results in a heightened effect when both components are shielded from degradation by durlobactam [90]. In terms of MIC, the introduction of imipenem has minimal impact on the effectiveness of sulbactam–durlobactam in combating drug-resistant strains of CRAB. An assessment of the 175 initial CRAB strains in the ATTACK trial revealed that the MIC50 and MIC90 values of sulbactam–durlobactam (2/4 and 4/4 mg/L, respectively) and imipenem–sulbactam–durlobactam (1/4 and 4/4 mg/L, tested in a 1:1 ratio of imipenem: sulbactam) were found to be comparable [82]. In contrast, the addition of imipenem to sulbactam–durlobactam in the RCT (*n* = 8, all with PBP3 mutations; sulbactam–durlobactam MIC range: 8/4–16/4 mg/L) resulted in a sulbactam–durlobactam MIC value below the CLSI breakpoint of 4/4 mg/L for seven out of eight of the strains tested.

When considered collectively, these in vitro findings indicate that the combination of a carbapenem with sulbactam–durlobactam in CRAB strains with reduced susceptibility to sulbactam–durlobactam, potentially due to PBP2 oversaturation caused by the carbapenem, may lead to enhanced potency. This phenomenon could be particularly relevant in the context of PBP3 mutations that affect sulbactam interactions. The data are more ambiguous in sulbactam–durlobactam-susceptible strains. While MICs are only slightly decreased with the inclusion of imipenem in this context, MIC assessment may not be the most suitable method to evaluate the effect of PBP-based interactions on efficacy. A recently published study demonstrated the combination of imipenem, sulbactam, and durlobactam to be highly effective in laboratory tests, even when MICs remained unchanged [90]. This combination displayed notable synergistic effects, resulting in enhanced antibacterial activity against sulbactam–durlobactam-resistant CRAB. Intriguingly, the synergy observed with imipenem–sulbactam (without durlobactam, due to the lack of carbapenemases) in a carbapenem-resistant isolate remained consistent without a reduction in MIC, thereby validating the hypothesis that the enhanced effect is primarily due to synergistic PBP interactions rather than the direct inhibition of OXA carbapenemases by imipenem. However, it should be noted that these experiments were conducted at levels significantly below those that would be achieved in actual clinical settings. Consequently, the clinical significance of these observations remains uncertain. Consequently, the existing in vitro findings are inadequate to ascertain the involvement of the carbapenem addition in the results observed in ATTACK. The influence of an additional carbapenem on both clinical results and emergence of antibiotic resistance in sulbactam–durlobactam-susceptible and PBP3-mutant CRAB strains remains uncertain and requires additional research.

In instances where clinical evidence is lacking, a comprehensive evaluation of the PK/PD association involving sulbactam–durlobactam and CRAB can offer valuable insights regarding the requirement of a carbapenem. Provided the PK/PD statistics are reliable and labeled dosing is used to achieve treatment levels, clinicians will be able to use sulbactam–durlobactam monotherapy with increasing confidence.

Firstly, dose-fractionation studies in a hollow-fiber infection model conducted using a CRAB strain with sulbactam and sulbactam–durlobactam MIC values of 16 and 4/4 mg/L, accordingly, determined that the 24 h area under the plasma concentration–time curve (AUC24h/MIC) is the most suitable durlobactam pharmacokinetic/pharmacodynamic (PK/PD) index [91].

Secondly, when evaluated in conjunction with sulbactam exposure, exhibiting an fT/MIC ratio of 85%, the study revealed that durlobactam AUC24h/MIC values of 13.8 and 24.2 were correlated with 1-log10 and 2-log10 CFU decreases, accordingly. The same CRAB strain was then selected to establish the durlobactam PK/PD threshold in a simplified in vitro infection simulation [91].

**Figure 2:**
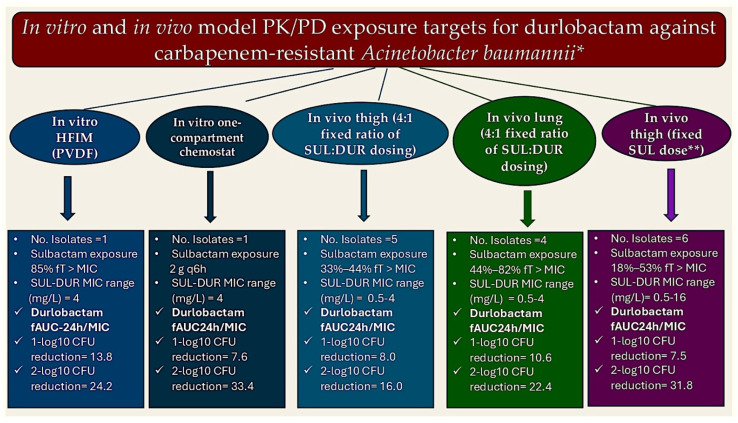
The figure illustrates the in vitro and in vivo PK/PD model exposure target values for durlobactam against carbapenem-resistant *Acinetobacter baumannii*. There are five models, which are distinguished by different colors. * AUC24h/MIC: area under the plasma concentration–time curve over 24 h; CFU: colony-forming unit; SUL: sulbactam; DUR: durlobactam; MIC: minimum inhibitory concentration; HFIM: hollow-fiber infection model; PVDF: polyvinylidene fluoride; ** in this study, the investigators employed a fixed sulbactam dosage to achieve fT > MICs ranging from 18% to 53%, while systematically adjusting durlobactam exposure levels to identify the durlobactam PK/PD target [54].

The investigative team established the durlobactam AUC24h/MIC target associated with activity in the context of simulated exposures representing 2 g of sulbactam every 6 h. They determined that AUC24h/MIC targets for durlobactam with an AUC24h/MIC of 7.6 and 33.4 are necessary for 1-log10 and 2-log10 CFU reductions [54] (Figure 2).

While these two in vitro tests generally align in terms of the extent of durlobactam exposure required to restore the 1-log10 kill with sulbactam (AUC24h/MIC of 7.6–13.8), it is important to note that these target values were derived under conditions of sulbactam exposures that may not fully reflect the actual clinical scenario. The durlobactam PK/PD target required to reinstate sulbactam activity is contingent on the sulbactam concentration (i.e., a higher sulbactam concentration necessitates a lower inhibitory threshold from durlobactam to attain stasis and 1-log10 decrease targets), implying that elevated sulbactam doses (2 g q6h instead of the stipulated 1 g q6h dose) and a potential enhancement in fT > MIC exposure beyond the capabilities of the isolate MIC (fT > MIC of 85%) restrict the generalizability of these findings. Furthermore, it should be noted that using a single CRAB strain to create both simulations may not adequately demonstrate variability in the PK/PD relationship that could exist among a broader range of susceptibilities. Therefore, the clinical usefulness of the findings from these in vitro tests to establish the best durlobactam dosage level is uncertain.

Further research was conducted into PK/PD models by administering plasma injections to murine neutropenic thigh and lung infection test animals. The aim was to pinpoint suitable treatment options for sulbactam, when administered in conjunction with durlobactam, and durlobactam, when administered in tandem with sulbactam, in combating CRAB [91]. In the study, sulbactam was co-administered with a single sulbactam-susceptible isolate, and a fixed 4:1 dose of sulbactam–durlobactam was administered against multiple CRAB isolates. The results demonstrated that a sulbactam fractional inhibitory concentration (fT) > minimal MIC of 29.9% and 38.2% in the thigh tissue infection model, and 41.2% and 53.5% in the lung infection model, were correlated with a 1-log10 and 2-log10 kill, accordingly. In the 4:1 dose calibration framework, the assessment of durlobactam concentration thresholds to reinstate sulbactam efficacy in counteracting CRAB strains revealed a fAUC24h/MIC of 8.0 and 16.0 in the thigh tissue infection model and 10.6 and 22.4 in the lung model, resulting in 1-log10 and 2-log10 kill, aligning closely with in vitro modeling predictions. The fixed 4:1 ratio was selected to align with the model’s predicted antibacterial activity in vitro; however, this approach may not be directly applicable to clinical settings, as the actual administration of these drugs is in a 1:1 ratio rather than the model’s 4:1 prediction [91]. It is evident that whilst all evaluated models positioned the durlobactam fAUC24h/MIC target within the range of 10, with the objective of achieving 1-log10 reductions in conjunction with sulbactam, concerns regarding the clinical applicability of this estimation persisted.

Nevertheless, further analysis of the neutropenic thigh experiment yielded valuable clinical insights regarding the durlobactam concentrations required to restore sulbactam efficacy. In this experiment, the researchers administered a fixed dose of sulbactam to achieve fT > MIC exposures ranging from 18% to 53% against six strains with MICs ranging from 0.5/4 to 16/4 to sulbactam–durlobactam. These concentrations are both relevant to clinical practice and align with the fT > MIC target of 50% that was used to assess the joint probability of target attainment [91]. The methodology employed by the researchers enabled the identification of durlobactam fAUC24h/MIC values of 7.5 and 31.8 as the key targets for achieving 1-log10 and 2-log10 kill with sulbactam, correspondingly. It is important to note that the target values were almost equivalent throughout the tested sulbactam–durlobactam MIC values, indicating a proportional relationship between dosage and sensitivity.

**Figure 3:**
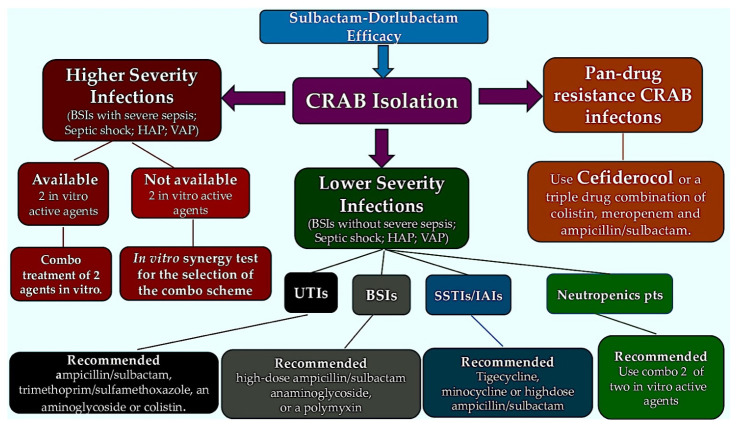
The proposed decision-making process for carbapenem-resistant *Acinetobacter baumannii* infections (CRAB) is outlined herewith. The color of the box is intended to represent the severity of the condition and the specific treatment selected. With regard to in vitro synergy, the optimal combinations are as follows: an aminoglycoside or polymyxin in combination with high-dose ampicillin sulbactam, high-dose tigecycline, or high-dose minocycline. Abbreviations; BSI, bloodstream infections; CRAB; carbapenem-resistant *Acinetobacter baumannii* infection; HAP, hospital-acquired pneumonia; IAI, intra-abdominal infection; SSTI, skin and soft tissue infection; UTI, urinary tract infection; VAP, ventilator-associated pneumonia.

Taking these preclinical PK/PD findings into consideration, a 50% fT > MIC for sulbactam and an fAUC24h/MIC of 10 for durlobactam have been identified as the PD targeting parameters for CRAB and have thus been incorporated into PTA assessments [91]. When evaluating the differential epithelial lining fluid (ELF) penetrance of sulbactam and durlobactam in mice and humans [91,92,93], and population PK data from eight Phase I–III clinical trials [94,95], Monte Carlo simulated analyses were conducted to determine the likelihood of target achievement. These analyses were performed using the package insert dosage across renal function categories. While these findings are not currently available in a published version, a summary of the results suggests that ≥90% PTA is attained for all dosing regimens adjusted for renal function for sulbactam–durlobactam MIC values up to the threshold of 4/4 mg/L in both plasma and ELF [49,94]. At an MIC (minimal inhibitory concentration) of 8/4 mg/L, PTA (protein trough concentration) falls significantly below 90% in patients with normal renal function. These findings indicate that sulbactam–durlobactam monotherapy should not be relied upon in cases of isolates with potential PBP3 variants demonstrating low-level non-susceptibility. In fact, an evaluation of ELF concentrations in the HFIM revealed that 1 g sulbactam q6h (32% fT > MIC) and 1 g durlobactam q6h (estimated fAUC24h/MIC of 19.5) did not attain 24 h bacterial stabilization in two PBP3-mutant CRAB strains with sulbactam–durlobactam MICs of 8/4 mg/L [54]. It is noteworthy that the combination of 1 g meropenem q6h or 1 g imipenem q6h to both isolates resulted in a log10-CFU decline that was comparable to that achieved by sulbactam–durlobactam alone when tested versus a CRAB strain with a sulbactam–durlobactam MIC of 0.5 mg/L. This equates to an order of 3- to 5-log10 CFU decrease from the initial level (Figure 3).

### 3.4. Efficacy of Sulbactam–Durlobactam in Treating Bacteria Other than A. baumannii

As was stated prior, direct PBP2 inhibition is demonstrated by durlobactam as a result of its intrinsically antibacterial efficacy against *E. coli* and other Enterobacterales. The treatment of NDM-producing *E. coli*, an increasingly prevalent global threat, is one clinical scenario in which this may be important. When examined in relation to 32 carbapenemase-producing Enterobacterales strains, encompassing MBL-producing isolates, the MIC levels of durlobactam varied from 0.125 to 8 mg/L [81]. According to current recommendations, cefiderocol or a combination of aztreonam and ceftazidime–avibactam should be considered for treatment of strains exhibiting this specific resistance genotype (Table 1). These medications primarily target PBP3. PBP3 insertions, when present in NDM-producing *E. coli*, have been shown to reduce the binding affinity of both aztreonam and cefiderocol, thereby reducing susceptibility [96,97]. This is in spite of the fact that PBP3 insertions offer stability against NDM-mediated hydrolysis. A large and increasing body of supporting research indicates that *E. coli* isolates which secrete NDM have alarmingly high levels of non-susceptibility to cefiderocol and avibactam–aztreonam, with respective susceptibility levels recorded as minimally 34% and 80% [98]. The reduced activity of aztreonam–avibactam can be partially explained by PBP3 insert mutations. In addition, co-harbored CMY variants further contribute to avibactam’s inability to fully protect aztreonam from hydrolysis [96]. Although durlobactam cannot suppress NDM enzyme activity, it is resistant to hydrolysis. Its PBP2-mediated in vitro efficacy makes it a promising adjunctive therapy for strains with decreased sensitivity to cefiderocol or avibactam–aztreonam, similar to the advantage of adding a carbapenem to sulbactam–durlobactam for CRAB strains with increased sulbactam–durlobactam MICs resulting from PBP3 alterations. Research has demonstrated that durlobactam can reliably maintain MIC levels within the range of 0.125 to 2 mg/L when facing NDM-producing *E. coli*, with MIC50 and MIC90 values recorded at 0.25 mg/L and 1 mg/L, correspondingly [98,99].

It is important to note that there are possible issues regarding the clinical application of durlobactam, given the frequent occurrence of resistant strains identified with other PBP2 beta-lactamase-inhibiting agents, such as mecillinam [100]. Nevertheless, using this in conjunction with other medications, such as cefiderocol or aztreonam–avibactam, and their associated PBP3 interactions, may help to prevent the emergence of resistance and maintain effectiveness. Table 1 shows that development is ongoing for cefepime–zidebactam, an equally promising candidate that has shown strong in vitro and in vivo effectiveness against NDM-producing bacteria with PBP3 inserts. This is despite the fact that cefepime is unstable when exposed to NDM and has reduced binding when PBP3 mutations are present, likely due to zidebactam’s capacity to hinder PBP2 [101]. While there is a lack of relevant clinical evidence concerning durlobactam and NDM-producing *E. coli*, a supplementary review of the ATTACK study looked at how well sulbactam–durlobactam, when used with imipenem, worked in 12 individuals with a variety of bacterial infections that included CRAB and imipenem-resistant (MIC ≥ 4 mg/L) *K. pneumoniae* [102]. In vitro tests showed that durlobactam was effective in fighting off 11/12 bacteria (with an MIC range of 1–8 mg/L) and lowered the imipenem MIC to 0.5–2 mg/L in 10/12 cases. The 28-day mortality rate in this group of individuals was 2 out of 12 (17%), and the clinical cure rate was 9 out of 12 (75%), both of which are comparable to the outcomes observed in subjects with monomicrobial CRAB infections. It is unclear what drove the effectiveness of durlobactam in these patient groups: whether it was due to the medication’s direct antibacterial properties, its ability to inhibit beta-lactamase and restore the activity of imipenem, or a combination of these mechanisms. Given the small sample size, it is also possible that the effectiveness was merely an incidental finding. It is too early to advocate for the use of durlobactam in these circumstances, but further investigation into its in vitro activity, PK/PD, and in vivo effectiveness versus Enterobacterales is necessary.

When it comes to *P. aeruginosa*, durlobactam has been observed to trigger alterations in morphology that could potentially affect its virulence. However, it has not been shown to exhibit standalone antibacterial efficacy in in vitro settings, a characteristic that has been noted in Enterobacterales [81]. Conversely, durlobactam could occupy a unique position among other DBOs as a BLI in certain MDR *P. aeruginosa* infections. In laboratory tests, durlobactam enhanced the activity of cefepime more strongly against certain types of bacteria (*P. aeruginosa*) than other antibiotics (vaborbactam, avibactam and relebactam). This is in line with its inhibition spectrum. As anticipated, durlobactam also increased the activity of meropenem and cefepime against bacteria that produce Class A, C and D beta-lactamases and carbapenemases [103] (Table 1). In the second stage of the ATTACK RCT outlined above, the research team examined the results for three cases in which sulbactam–durlobactam was administered alongside imipenem to address a polymicrobial infection triggered by CRAB and imipenem-resistant *P. aeruginosa*. The MIC threshold for imipenem was found to be 16 mg/L in all three individuals’ *P. aeruginosa* strains [102]. Adding durlobactam (4 mg/L) reversed the loss of in vitro efficacy observed in all three isolates (MIC 1–2 mg/L). The findings are comparable to those observed when relebactam is coupled with imipenem in the presence of imipenem-resistant *P. aeruginosa*. All three were alive. Two had a positive outcome. The third showed an unclear result.

New findings suggest that existing DBOs (avibactam, relebactam and durlobactam) could be used to treat ceftolozane-resistant strains of *P. aeruginosa* [104] (Table 1). This is due to mutations that increase the effectiveness of ampC-mediated hydrolysis of ceftolozane. When clinical *P. aeruginosa* strains that had grown resistant to ceftolozane–tazobactam were analyzed before and after treatment, it was found that ceftolozane–durlobactam caused the smallest change to the MIC from the starting point when compared to the other DBOs, avibactam and relebactam. Table 1 is in line with what was previously found about the more powerful effect of durlobactam in fighting specific PDC strains [104]. Although using durlobactam to increase the effectiveness of different beta-lactams in treating *P. aeruginosa* is not yet a viable option, healthcare professionals are advised to be informed of its possible advantages in specific clinical situations. Further investigation of durlobactam in such cases is necessary.

## 4. Future Direction

A review of the PK/PD data reveals that the sulbactam–durlobactam combination is likely to be effective in the treatment of CRAB cases with MIC values up to 4/4 mg/L. It is particularly important to add a carbapenem when sulbactam–durlobactam MICs fall somewhere in the 8/4 range (milligrams per liter). This is the best way to identify the most effective course of treatment. However, comparative evidence on the effectiveness of sulbactam–durlobactam for the treatment of CRAB is currently unavailable, both as a standalone treatment and in conjunction with a carbapenem. While the PK/PD guidelines support the exclusion of the carbapenem for susceptible strains, the available in vitro data demonstrate the added efficacy of a carbapenem in enhancing the effectiveness of sulbactam–durlobactam.

In addition, the current clinical data mainly assess the utilization of sulbactam–durlobactam in combination with imipenem. While it is feasible and potentially probable that the effectiveness exhibited in the ATTACK study was attributable to sulbactam–durlobactam alone, it is also conceivable that the impact of sulbactam–durlobactam—even if it proves effective on its own—was amplified by the incorporation of the carbapenem.

## 5. Conclusions

Prior to the authorization of sulbactam–durlobactam as the preferred treatment option in this clinical area, options for treating severe infections due to carbapenem-resistant *A. baumannii* were restricted. In vitro tests have confirmed the high level of effectiveness of sulbactam–durlobactam against carbapenem-resistant *A. baumannii*. Presently, instances of elevated resistance are rare, largely attributable to the widespread presence of MBL synthesis.

Despite the paucity of head-to-head comparative clinical evidence available to inform decision-making, sulbactam–durlobactam has demonstrated superiority to cefiderocol for the treatment of carbapenem-resistant *Acinetobacter baumannii* infections in comparable randomized clinical trials. The carbapenem-resistant *A. baumannii* PD objectives of 50% fT > MIC for sulbactam and fAUC24h/MIC of 10 for durlobactam are substantiated by consensus derived from in vitro and in vivo PK/PD evaluations, and the results of initial MCS indicate that the PTA of designated administered quantities up to the identified resistance threshold of 4/4 mg/L is appropriate.

Although the in vitro evidence unequivocally supports the efficacy of carbapenem combo regimens when the MIC indicates minimal sulbactam–durlobactam resistance, the role of combo therapeutics with a carbapenem in sensitive strains remains ambiguous. Until more clinical evidence is provided, we are in agreement with the IDSA’s recommendation for carbapenem combination therapeutic treatment for those suffering from serious infections caused by the presence of carbapenem-resistant *A. baumannii* bacteria.

Durlobactam is a potent inhibitor of PBP2 in Enterobacterales and a DBO BLI with significant Class A, C, and D enzyme inhibitory activity. It has the potential to address specific therapeutic needs as a life-saving intervention for cases involving NDM-producing *E. coli* with PBP3 integrations or as a complementary therapeutic option to re-establish beta-lactam efficacy in combating DTR *P. aeruginosa*. However, additional research is undoubtedly required to fully substantiate the efficacy of either of these proposed courses of action.

## 6. Limitation

One potential shortcoming of this narrative therapy study is the absence of substantial data from multicenter randomized controlled trials (RCTs) on cefiderocol monotherapy. Nonetheless, earlier research has shown that cefiderocol is at least as effective as high-dose, extended-infusion meropenem monotherapy in reducing all-cause mortality on day 14. This was observed in critically ill patients with hospital-acquired pneumonia caused by a wide range of Gram-negative bacteria, including *A. baumannii*, *P. aeruginosa*, and Enterobacterales. Data supporting optimal antibiotic therapy, even for patients with non-pulmonary infections caused by Gram-negative bacteria, must be derived from randomized multicenter studies comparing different antibiotics.

## Figures and Tables

**Table 1:** Therapeutic review searching strategies.

Items	Specification
Date of search (specified to date, month and year)	From June 2025 to December 2025
Databases and other sources searched	PubMed, MEDLINE, Embase, and the Cochrane Library
Search terms used (including MeSH and free text search terms and filters)	sulbactam–durlobactam; carbapenem-resistant *Acinetobacter baumannii*; *Acinetobacter baumannii* infection; multidrug-resistant *Acinetobacter baumannii–calcoaceticus* complex; ventilator-associated bacterial pneumonia; hospital-acquired bacterial pneumonia combined with World Health Organization”, “Central Disease Control”, and “Federal Drug Administration”
Timeframe	Up to June 2025
Inclusion and exclusion criteria (study type, language restrictions, etc.)	English language; inclusion criteria: the manuscripts under consideration here all concern cases of carbapenem-resistant *Acinetobacter baumannii* infections
Selection process	One author independently selected articles after screening for duplicates.

**Table 2:** Recommended dose of beta-lactams for adults with antimicrobial-resistant infections and normal renal and hepatic function in the treatment of *A. baumannii* and bacteria other than *A. baumannii* *,^#^.

**Ampicillin–sulbactam**	The recommended total daily dose of sulbactam is 9 g, to be administered via one of the following regimens: ♦ 9 g of ampicillin–sulbactam (6 g ampicillin, 3 g sulbactam) intravenously every 8 h, infused over 4 h ♦ 27 g of ampicillin–sulbactam (18 g ampicillin, 9 g sulbactam) intravenously as a continuous infusion over 24 h
**Ceftazidime–avibactam**	The dosage of 2.5 g is to be administered intravenously every 8 h, over a period of 3 h.
**Ceftazidime–avibactam PLUS aztreonam**	The following medications should be infused over 3 h. ♦ **Ceftazidime–avibactam:** 2.5 g IV every 8 h, administered via Y-site ♦ **Aztreonam:** 2 g IV every 8 h, administered via Y-site
**Ceftolozane–tazobactam**	♦ **Uncomplicated cystitis:** 1.5 g intravenously (IV) every 8 h, administered over 1 h. ♦ **All other infections**: 3 g IV every 8 h, administered over 3 h.
**Cefiderocol**	The dosage of 2 g should be administered intravenously every 8 h, with the infusion being completed over a 3 h period. For patients with a creatinine clearance rate of at least 120 mL per minute, the dosage should be reduced to 2 g every 6 h, with the infusion being administered over a 3 h period.
**Cefepime**	♦ **For uncomplicated cystitis:** The recommended dosage is 1 g intravenously every 8 h, infused over 30 min. ♦ **For all other infections:** The patient should receive 2 g intravenously every 8 h, over a period of 3 h.
**Imipenem–cilastatin–relebactam**	The dosage is 1.25 g intravenously, administered over a 30 min period.
**Imipenem–cilastatin**	♦ **For uncomplicated cystitis**: The dosage is 500 mg intravenously every 6 h, infused over 30 min. ♦ **For all other infections**: The dosage of 500 mg should be administered intravenously every 6 h, with the infusion being completed over a period of 3 h, if this is deemed a viable option.
**Meropenem–vaborbactam**	The patient should receive 4 g intravenously every 8 h, over a period of 3 h.
**Meropenem**	♦ **For uncomplicated cystitis:** The recommended dosage is 1 g intravenously every 8 h, infused over 30 min. ♦ **For all other infections:** The recommended dosage is 2 g intravenously, administered over a period of 3 h (if feasible).
**Sulbactam–durlobactam**	**Sulbactam and durlobactam** should be administered intravenously at a dose of 1 g each, with a total dose of 2 g, every 6 h, over a 3 h infusion. For patients with a creatinine clearance of at least 130 mL per minute, the dosing schedule is 1 g of sulbactam and 1 g of durlobactam, administered intravenously every 4 h, over a 3 h infusion.

Abbreviations: * The dosing recommendations are limited to those specified in the IDSA AMR Treatment Guidance document, which covers organisms and infectious syndromes. ^#^ The dosing recommendations for several agents may not align with the dosing guidelines set out by the United States Food and Drug Administration.

## Data Availability

No new data were created or analyzed in this study.
